# The impact of COVID-19 on hepatitis B and C virus prevention, diagnosis, and treatment in Bangladesh compared with Japan and the global perspective

**DOI:** 10.1186/s12913-023-10138-x

**Published:** 2023-10-23

**Authors:** Md Razeen Ashraf Hussain, Mohammad Ali, Aya Sugiyama, Lindsey Hiebert, M. Anisur Rahman, Golam Azam, Serge Ouoba, Bunthen E, Ko Ko, Tomoyuki Akita, John W. Ward, Junko Tanaka

**Affiliations:** 1 Department of Epidemiology, Infectious Disease Control and Prevention, Graduate School of Biomedical and Health Sciences, Hiroshima University, 1-2-3, Kasumi, Minami-Ku, Hiroshima-Shi, 734-8551 Japan; 2 Department of Hepato-Biliary-Pancreatic Surgery & Liver Transplant, BIRDEM General Hospital, Dhaka, Bangladesh; 3 National Liver Foundation of Bangladesh, Dhaka, Bangladesh; 4 Coalition for Global Hepatitis Elimination, The Task Force for Global Health, Decatur, GA USA; 5 Department of Gastrointestinal Hepatobiliary & Pancreatic Disorders (GHPD), BIRDEM General Hospital, Dhaka, Bangladesh; 6 Unité de Recherche Clinique de Nanoro (URCN), Institut de Recherche en Science de La Santé (IRSS), Nanoro, Burkina Faso; 7 National Payment Certification, National Social Protection Council, Ministry of Economic and Finance, Phnom Penh, Cambodia

**Keywords:** HBV, HCV, COVID-19, Impact, Cross sectional study, Comparative analysis, Bangladesh

## Abstract

**Aim:**

This study aimed to assess the effect of COVID-19 on hepatitis-related services in Bangladesh and compared the situation with same study conducted in Japan and globally.

**Methods:**

We conducted an online cross-sectional questionnaire survey among the clinicians of four societies associated with liver disease in Bangladesh from October to December 2022. The questionnaire included the same questions as a survey conducted in Japan and globally.

**Results:**

A total of 83 clinicians from 8 divisions in Bangladesh participated; 66.3% were heads of departments/institutions. Except for HCV treatment initiation, more than 30% of clinicians reported a 76–99% decline in all services. Compared to Japan and the global survey, there was a significantly higher decline in all HBV and HCV services in Bangladesh. To resume services back to pre-COVID-19 levels, Patient anxiety and fear (Bangladesh Survey: 80.7% vs Japan Survey: 67.4% vs Global Survey: 37.9%, *p* < 0.0001), loss of space due to COVID-19 (Bangladesh Survey: 63.9% vs Japan Survey: 34.7% vs Global Survey: 19.4%, *p* < 0.0001) were the main challenges. As part of the mitigation strategy, usage of telemedicine (Bangladesh Survey: 83.1% vs. Japan Survey: 67.3% vs Global Survey: 78.6% *p* < 0.0001), COVID-19 benefits, such as increased laboratory testing platforms (Bangladesh Survey: 77.1% vs Japan Survey: 17.9% vs Global Survey: 41.8%, *p* < 0.0001) was reported significantly higher in Bangladesh than in Japan and global survey.

**Conclusion:**

All the services-related to HBV and HCV were highly affected during greatest impact month of COVID-19 in Bangladesh and the decline level was higher than Japan and global survey. Repeated countermeasures of COVID-19 and constrained healthcare-system were the probable reasons in Bangladesh. Positive impact resulting from COVID-19 countermeasures should be utilized in the national hepatitis program in Bangladesh.

**Supplementary Information:**

The online version contains supplementary material available at 10.1186/s12913-023-10138-x.

## Background

In 2019, the World Health Organization (WHO) estimated 296 million people were living with hepatitis B virus (HBV) and 58 million were living with hepatitis C virus (HCV), including 1.5 million new infections in both cases [1]. Combating hepatitis is thus one of the Sustainable Development Goals (SDG 3.3) The WHO has set a goal of eliminating HBV and HCV by 2030 [2, 3]. To achieve this goal, large-scale screening and treatment are required [2, 3]. Bangladesh is a developing country in South Asia with a large population estimated 169 million. Based on the studies from around 2000’s, the country had an intermediate prevalence 5.5%-7.6% HBV infection [4–9]. The prevalence of HCV was around 0.5- 1% which was much lower than HBV [4, 10, 11]. In Bangladesh, the Expanded Program of Immunization (EPI) from 2003, with a coverage rate of more than 97% and free screening offered by government hospitals and philanthropic organizations like the National Liver Foundation of Bangladesh (NLFB) during awareness campaign, subsidy for underprivileged people have significantly contributed to the rise in diagnoses and detection of HBV and HCV [10, 12–14]. In private clinics and hospitals, screening and testing are not free of charge but treatment for hepatitis is less expensive in Bangladesh than in many other countries because of locally produced generic anti-HBV and anti-HCV medications [12, 15]. So, continuous approaches by the government, private sector, non-governmental organizations (NGOs) and various organizations have been contributing to improving the situation to reach the 2030 hepatitis elimination target [10, 13–17]. As a result, from the recent government report and studies (2018–2022), the prevalence of HBV was 4–4.5% and that of HCV was 0.5–0.6% in the general population of Bangladesh [12, 13, 15, 17–19]. However, during the progress of hepatitis elimination in Bangladesh, COVID-19 pandemic occurred which was declared by World Health Organization (WHO) and the first case detected in Bangladesh on March 8, 2020 [20]. Till the survey period, about 2 million cases and about 30,000 deaths were reported from the COVID-19 pandemic in Bangladesh [21]. Although the mortality rate is low so far (1.4%), the whole healthcare system and public health in developing countries like Bangladesh has been reduced due to the limited budget and several countermeasures such as lockdowns, shutdown affected by the COVID-19 pandemic [22–24]. The Coalition for Global Hepatitis Elimination (CGHE), a program of the Taskforce for Global Health conducted a global survey in 2020 among 44 countries on the disruption of HBV and HCV screening and testing due to COVID-19 pandemic The survey revealed a decline and reduction in HBV and HCV treatment, screening and testing at different levels in different WHO regions [25]. The same study was conducted in developed country Japan and a 1–25% decrease in screening and confirmatory testing was reported by clinicians [26]. Such reported declines suggested understanding the situation the impact of COVID-19 on hepatitis in intermediate endemicity with a large population in lower middle income country like Bangladesh. As the same study was conducted in Japan and global survey, it will supplement to understand the situation in Bangladesh. Additionally, the comparison of the situation of COVID-19 effect on hepatitis related services between Japan who have progressed tremendously on HBV and on the track of HCV elimination with Bangladesh who was not on the track towards HBV and HCV and progressing towards hepatitis elimination will illustrate a broader scenario [27]. Considering all the above, this study aimed to assess the effect of COVID-19 on hepatitis B virus and hepatitis C virus-related services in lower middle income country Bangladesh and compare the situation with Japan survey and global survey.

## Methods

### Study design and settings

This study was an online cross-sectional survey developed in Microsoft Forms. This study was conducted by the Hepatitis Policy Research Project (Epidemiology Group) of the Ministry of Health, Labor and Welfare (MHLW) of Japan and the Coalition for Global Hepatitis Elimination (CGHE) of the Task Force for Global Health, USA in collaboration with the National Liver Foundation of Bangladesh (NLFB). The questionnaire was adapted from a global survey developed by the Task Force for Global Health and similar survey was conducted in Japan from same questionnaire [25, 26]. The survey included a total of 49 questions to assess the impact of the COVID-19 pandemic on access to HBV and HCV services and the program response to service delivery (Supplementary Table 1). The survey areas consisted of (i) basic information, (ii) HBV and HCV-related service delivery during COVID-19, (iii) challenges in recovering to pre-COVID-19 and COVID-19 levels and mitigation strategies, (iv) clinical involvement in the response to COVID-19, and (v) potential benefits of the response to COVID-19 for the hepatitis system. Several questions were added, modified, and rearranged based on the Bangladesh context. Electronic versions of the questionnaire were developed in both English and Bengali.

The survey targeted clinicians belonging to four societies dealing with liver diseases, namely the National Liver Foundation of Bangladesh (NLFB), the Hepatology Society Dhaka, the Association for the Study of Liver Diseases, and the Bangladesh Gastroenterology Society. The web link to the questionnaire was distributed by the NLFB via e-mail to all members of the above-mentioned societies. The survey was conducted from 23 October 2022 to 20 December 2022. The results of same questions were compared with Japan survey and global survey. In Japan survey a total 196 of clinicians participated which was conducted in 2021 and the global survey was conducted in 2020 in 44 countries where a total of 103 clinicians participated [25, 26]. Japan survey was conducted among hepatologists from Japan Society of Hepatology (JSH) members and the global survey conducted by CGHE and questionnaire distributed through their web among hepatologists all around the world. The questionnaire design was similar in both studies [25, 26].

### Survey analysis

Microsoft Excel for Microsoft 365 MSO (version 2112 build 16.0.14729.20254) was used to shop survey responses. Statistical analyses were performed using JMP15.0 software (SAS Institute Inc, Cary, NC, USA). Descriptive analyses of participant background characteristics and responses were performed; frequencies and percentages were calculated and reported. Wilcoxon signed rank test was performed to compare the difference between decline level of HBV and HCV related Services in Bangladesh. Chi-square tests were performed to compare categorical data between the Bangladesh survey, Japan survey and global survey result [25, 26], and p values less than 0.05 were considered statistically significant.

### Ethical consideration

This was a cross-sectional survey for which ethical approval was obtained for data collection and analysis. The epidemiological research ethics committees at Hiroshima University (approval number E-2021–2530-01) and the ethics committee of National Liver Foundation of Bangladesh (approval number: 001/Ethics/2022) has provided the approval for this study. All the study activities were conducted by relevant guidelines and regulations and no information on patients or their personal information on their health was collected.

## Results

### Survey respondents

**Fig. 1:**
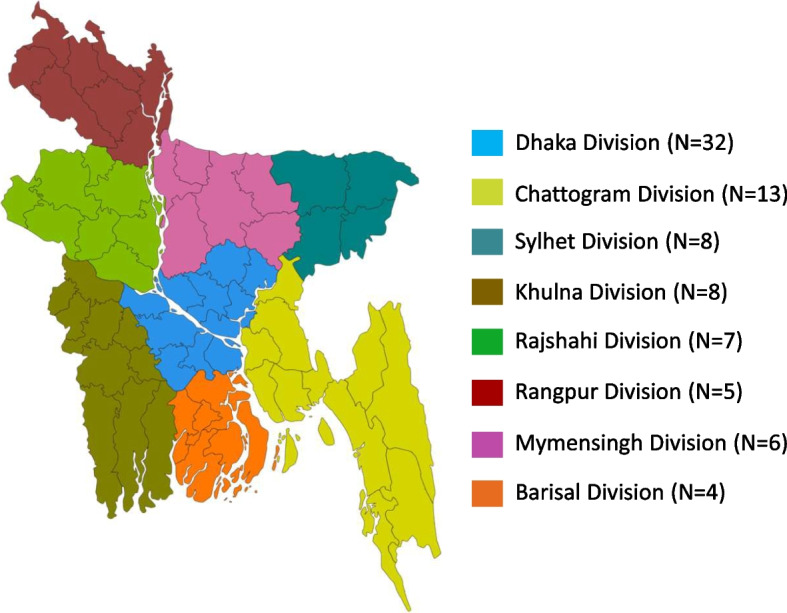
Participants distribution of Bangladesh survey based on Division. This figure represents distribution of 83 clinicians from NLFB, the Hepatology Society Dhaka, the Association for the Study of Liver Diseases, and the Bangladesh Gastroenterology Society division of Bangladesh. Blue color represents Dhaka division, lime color represents Chattogram division, teal color represents Sylhet division, gold color represents Dhaka division, Dark green color represents Rajshahi division, Dark red color represents Rangpur division, pink color represents Mymensingh division and orange color represents Barisal division

Of 300 total members of NLFB, the Hepatology Society Dhaka, the Association for the Study of Liver Diseases, and the Bangladesh Gastroenterology Society, a total of 83 clinicians responded to the survey. A total of 97.6% of clinicians were directly involved in screening, testing and treatment related to HBV and HCV. Most of the participants were from Dhaka division (38.6%) and Chattogram division (15.7%) (Fig. 1). About the participants’ specialty, 44.6% of clinicians were gastroenterologists and 42.2% were hepatologists. Among them 66.3% of participants were head of a department related to liver disease or head of hepatitis control at their institutions. From total 45.8% belonged to medical college and 27.7% were from private hospital (Table 1).

**Table 1:** Affiliated institution/facility of the clinicians in Bangladesh

Clinicians’ affiliated institution/facility	%
University Hospital (*N* = 7)	8.4%
Public Hospital (*N* = 11)	13.3%
Medical College (*N* = 38)	45.8%
Private Hospital (*N* = 23)	27.7%
Clinic (*N* = 3)	3.6%
Others (*N* = 1)	1.2%
Total *N* = 83	100%

### HBV and HCV related services

**Fig. 2:**
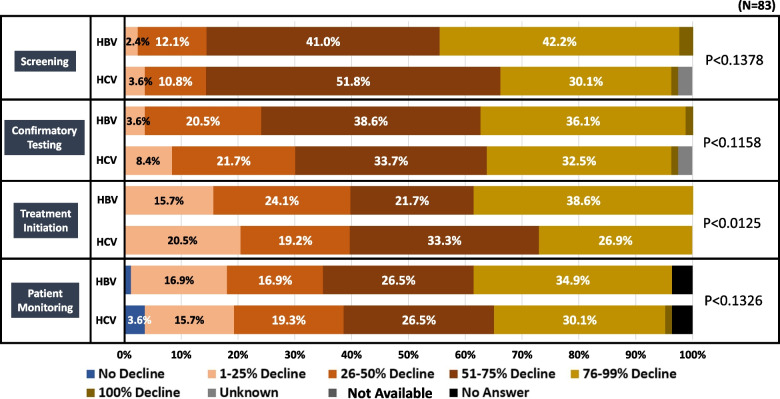
The decline level of HBV and HCV related services in Bangladesh during highest impact month of COVID-19. This figure represents the level of decline reported on HBV, HCV and HCC screening, treatment initiation and patient monitoring from the impression of the clinicians participated in Bangladesh survey. The impression was given based on greatest impact month of COVID-19 in Bangladesh till survey period (2022). HBV: Hepatitis B Virus, HCV: Hepatitis C Virus, HCC: Hepatocellular Carcinoma COVID-19: Coronavirus disease 2019

In the month of greatest impact from COVID-19, nearly every level of HBV and HCV screening, diagnosis, and treatment showed a decline from the levels prior to COVID-19. From a total of 42.2%, 38.6% and 34.9% reported a decline of 76–99% in screening, treatment initiation, and patient monitoring of HBV, respectively. On the other hand, 51.8%, 33.7% and 33.3% reported a 51–75% decline in screening, treatment initiation, and patient monitoring of HCV, respectively. However, except for treatment initiation (*p* < 0.0125), there was no significant difference in declines between HBV and HCV related services (Fig. 2).

**Fig. 3:**
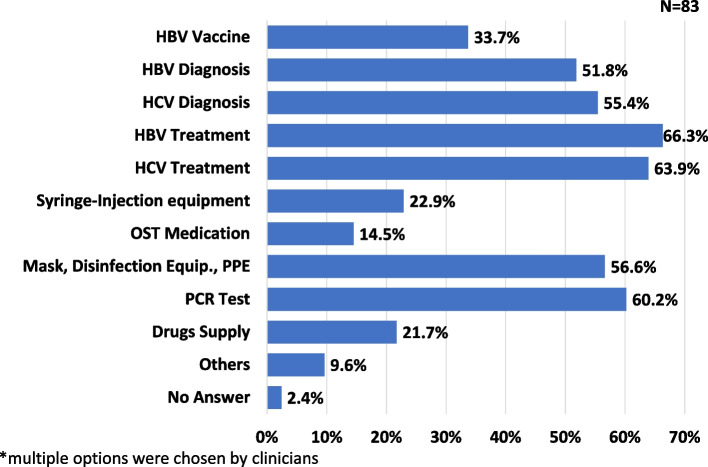
Reported supply chain disruption on hepatitis related services in Bangladesh. This figure represents the reported supply chain disruption during greatest impact month of COVID-19 related to COVID-19 from participated clinician in Bangladesh survey. the participating clinicians were allowed to select multiple options. COVID-19: Coronavirus disease 2019

A decline of 76–99% on HCC treatment initiation and a decline of 51–75% on monitoring of HCC patients were reported by 27.1% and 28.9% of clinicians, respectively. (Supplementary Fig. 1) A 51–75% decline in patients who were monitored for Sustained Virological Response 12 (SVR) treatment of HCV was answered by 31.3%. In addition, a 76–99% decline in treating drug addiction was reported by 34.9%. Supply chain disruption related to HBV treatment (66.3%), HCV treatment (63.9%) and PCR testing (60.2%) were mostly reported by the clinicians in Bangladesh survey (Fig. 3). Due to COVID-19, 84.3% of clinicians reported on cancellation on hepatitis management related meetings in Bangladesh.

### Other services deferred during COVID-19

Among diagnosis related services, gastrointestinal endoscopy (90.4%), liver biopsy (80.7%) and imaging (75.9%) were highly deferred during the greatest impact month of COVID-19. Regarding awareness related program, lecture for public awareness (88.0%) and lecture for patients (86.8%) were highly deferred reported by the clinicians in Bangladesh survey. Referral of patients from other departments (92.8%) and prescription interval (90.4%) were reported decline in the greatest impact month of COVID-19 (Table 2).

**Table 2:** Deferring for Liver related diagnosis and other services in Bangladesh and Japan

Aspect^a^	Defer during greatest Impact month of COVID-19	Survey^a^	Yes (%)	No (%)	Don’t Know (%)	NA (%)	No Answer (%)	*p*-value
**Diagnosis Related**	Defer Imaging (^c^)	Bangladesh (*N* = 83)	75.9	20.5	2.4	1.2	0.0	0.0261
		Japan^b^ (*N* = 196)	65.8	29.6	0.0	4.6	0.0	
	Defer Laboratory Testing	Bangladesh (*N* = 83)	66.3	28.9	2.4	1.2	1.2	0.0703
		Japan^b^ (*N* = 196)	68.4	27.6	0.0	4.1	0.0	
	Defer HCC Screening	Bangladesh (*N* = 83)	65.1	30.1	3.6	0.0	1.2	0.0064
		Japan^b^ (*N* = 196)	55.1	39.8	0.0	4.6	0.5	
	Gastrointestinal Endoscopy	Bangladesh (*N* = 83)	90.4	1.2	6.0	0.0	2.4	0.0001
		Japan^b^ (*N* = 196)	87.2	8.7	0.0	3.1	1.0	
	Liver Biopsy	Bangladesh (*N* = 83)	80.7	1.2	13.3	2.4	2.4	< 0.0001
		Japan^b^ (*N* = 196)	43.4	42.9	0.0	13.3	0.5	
**Public Awareness and lectures**	Nutritional Guidance	Bangladesh (*N* = 83)	55.4	31.3	12.1	0.0	1.2	< 0.0001
		Japan^b^ (*N* = 196)	38.8	49.0	0.0	12.2	0.0	
	Lecture for Patients	Bangladesh (*N* = 83)	86.8	4.8	1.2	1.2	6.0	< 0.0001
		Japan^b^ (*N* = 196)	55.6	10.7	0.0	33.2	0.5	
	Public Lecture for awareness	Bangladesh (*N* = 83)	88.0	0.1	0.0	0.0	0.0	< 0.0001
		Japan^b^ (*N* = 196)	55.6	27.9	0.0	26.0	0.5	
**Hospital Visit Related**	Referral of Patients from others department	Bangladesh (*N* = 83)	92.8	1.2	2.4	0.0	3.6	< 0.0001
		Japan^b^ (*N* = 196)	33.7	54.6	0.0	11.2	0.5	
	Extend Hospital Visit	Bangladesh (*N* = 83)	74.7	21.7	1.2	0.0	2.4	0.0074
		Japan^b^ (*N* = 196)	65.0	28.6	0.0	6.1	0.0	
	Prescription interval	Bangladesh (*N* = 83)	90.4	8.4	1.2	0.0	0.0	< 0.0001
		Japan^b^ (*N* = 196)	51.0	40.3	0.0	6.1	2.6	

^a^Questions on those aspects were not included in global survey

^b^ Japan survey was conducted in 2021 [26]

^c^Including HCC screening

### Challenges to resume services and mitigation strategies

**Fig. 4:**
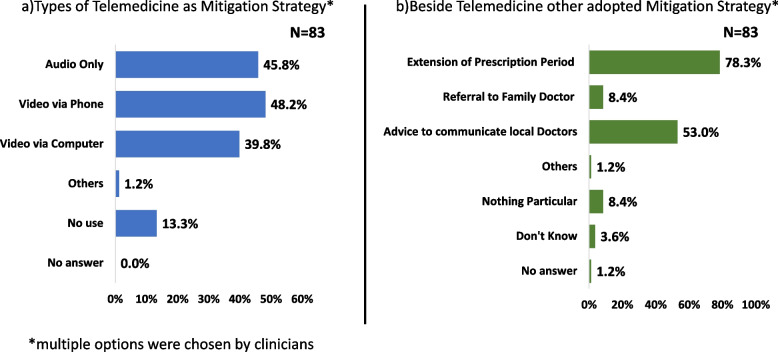
Mitigation strategy a) telemedicine and b) other strategies adopted beside telemedicine in Bangladesh. This figure represents the mitigation strategies by the clinicians participated in Bangladesh survey.  First part is presenting the types of telemedicine and the second part is representing other strategies which were adopted by clinician beside telemedicine. On both cases, the participated clinicians were allowed to select multiple options on telemedicine and beside telemedicine. COVID-19: Coronavirus disease 2019

Challenges to resume services to pre-COVID-19 level such Patient anxiety and fear limited (80.7%) and loss of space due to COVID-19 (63.9%) were highly reported by the clinician in Bangladesh survey (Table 3). As part of the mitigation strategy, telemedicine was commonly reported to use by the clinicians in the Bangladesh survey. Video via phone (48.2%) and audio call (45.8%) were commonly used (Fig. 4a). Beside telemedicine, other strategies were adopted, such as the extension of the prescription period (78.3%) and advice to communicate with local doctors (53.0%) by the clinicians in Bangladesh survey (Fig. 4b). Most of the clinicians in Bangladesh survey (37.4%) reported 51–75% usage of telemedicine instead of clinic visits (Fig. 6).

**Table 3:** Comparison of response of clinician on different area in Bangladesh, Japan and global survey

**Area**	**Issue/Facility**	**Bangladesh (*N* = 83)**	**Japan**^a^** (*N* = 196)**	**Global**^**b**^** (*N* = 103)**	***p*-Value**
**Challenges to resume services back to pre-COVID-19 level**	Patient Anxiety or Fear of COVID-19	80.7%	67.4%	37.9%	< 0.0001*
	Limited Availability Staff	41.0%	46.4%	17.5%	< 0.0001*
	Inadequate PPE	30.1%	12.2%	13.6%	0.0008*
	Loss of Space due to COVID-19	63.9%	34.7%	19.4%	< 0.0001*
	Loss of Staff due to COVID-19	34.9%	49.0%	6.8%	< 0.0001*
	Supply Chain Disruption	28.9%	13.8%	7.8%	0.0022*
	Loss of Funding with Direction to COVID-19	22.9%	16.3%	6.8%	0.008*
**Change to Control Infection**	Face Mask, Face Shield Used During Patients Encounter	95.2%	54.1%	82.5%	< 0.0001*
	Patient Routinely Check for COVID-19 Symptoms	60.2%	50.0%	47.6%	0.162
	Rigorous Cleaning of Surfaces	59.0%	54.1%	50.5%	0.4315
	Face Mask Required of Patients	67.5%	78.6%	55.3%	0.0004*
	Spacing of Patients Visit	59.0%	30.6%	45.6%	< 0.0001*
**Providing Care related to COVID-19**	Triage Outpatient	65.1%	29.6%	-	< 0.0001*
	COVID-19 Vaccination	21.7%	61.2%	-	< 0.0001*
	Sars-CoV-2 Testing	18.1%	51.5%	-	< 0.0001*
	Evaluate COVID-19 Sign/ Symptoms and Refer on Severity	56.6%	19.4%	-	< 0.0001*
	Manage All Aspects of Care for Patients with COVID-19	44.6%	19.4%	-	< 0.0001*
	Consultation on Liver or Infectious Disease Management	69.9%	43.9%	-	< 0.0001*
**Potential Benefits of COVID-19 on hepatitis care**	Increased Laboratory Platforms for HBV and HCV Testing	77.1%	17.9%	41.8%	< 0.0001*
	Improved Training of Primary Care in Infectious Disease	77.1%	35.7%	42.7%	< 0.0001*
	Improved Reporting of Laboratory Result	56.6%	19.9%	18.5%	< 0.0001*
	Improved Referral Networks for Complex Patients	34.9%	29.1%	22.3%	0.1168
	Improved Disease Surveillance	32.5%	14.3%	24.3%	0.0028*
	Improved Contact Tracing Which May Use for Hepatitis	44.6%	13.8%	25.2%	< 0.0001*
	Strengthening on Infectious Disease Control	69.9%	45.9%	-	0.0002*
	Raising Awareness on Infectious Disease Control	36.1%	44.4%	-	0.2766

^*^Statistically Significant, (-) question was not included in global survey

^a^ Japan survey was conducted 2021 [26]

^b^ Global survey was conducted 2020 [25]

### Response to COVID-19

To control infection changes such face mask/shield used during patients encounter (95.2%), glove-mask routinely used during patients encounter (77.1%) and spacing of patients visit (59.0%) were highly reported by the clinicians in Bangladesh survey (Table 3). Due to the emergence of COVID-19, in total, 95.2% of clinicians reported changing their activities. Providing care related to COVID-19, such as triage outpatient (65.1%) and consultation on liver or infectious disease management (69.9%) was answered by the clinicians in Bangladesh survey (Table 3).

### Potential benefits of the COVID-19 response to hepatitis elimination

Potential benefits such as increased laboratory platforms for HBV and HCV testing (77.1%), improved training of primary care in infectious disease testing and management (77.1%) and strengthening infectious disease control (69.9%) were highly reported by Bangladeshi clinicians (Table 3).

### Comparing Bangladesh survey situation with Japan and global survey

**Fig. 5:**
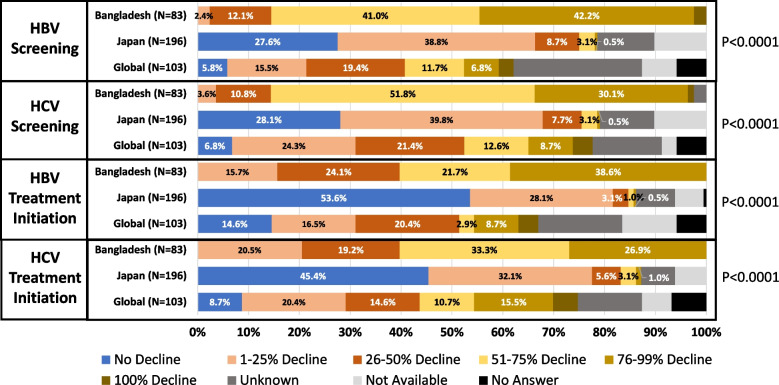
Comparison of decline level in Bangladesh, Japan and global survey on HBV and HCV related services during highest impact month of COVID-19. This figure shows the comparison of clinician’s impression in Bangladesh survey, Japan survey and global survey on the level of decline on HBV and HCV screening and treatment initiation and patient monitoring in respect of greatest impact month of COVID-19. Bangladesh survey conducted in 2022 where Japan and global survey conducted in 2021 and 2020, respectively. HBV: Hepatitis B Virus, HCV: Hepatitis C Virus, COVID-19: Coronavirus disease 2019

Most of the clinicians from Bangladesh survey reported any level of decline which was significantly higher than the clinicians participating in Japan and global survey for HBV (Bangladesh Survey: 100% vs Japan Survey: 51.1% vs Global survey: 56.3%, *p* < 0.0001) and HCV (Bangladesh Survey: 97.5% vs Japan Survey: 51.1% vs Global survey: 70.9%, *p* < 0.0001) screening. A similar result was observed for HBV (Bangladesh Survey: 100% vs Japan Survey: 32.7% vs Global survey: 52.4%, *p* < 0.0001) and HCV (Bangladesh Survey: 100% vs Japan Survey: 41.8% vs Global survey: 66.1%, *p* < 0.0001) treatment initiation (Fig. 5). Comparing with Japan survey, except for laboratory testing, all the other diagnostic services such as imaging, HCC screening, gastrointestinal endoscopy, liver biopsy were deferred significantly higher in Bangladesh than in Japan survey. For public awareness and lectures and hospital visit issue such referral of patients from other departments were deferred significantly higher in Bangladesh than in Japan (Table 2).

**Fig. 6:**
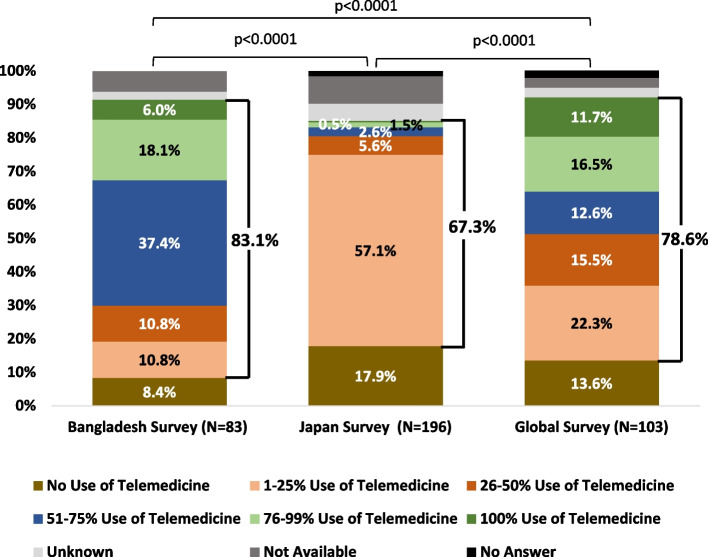
Comparison of usage of telemedicine in greatest impact month of COVID-19 in Bangladesh, Japan and Global survey. This figure shows the comparison of using telemedicine on greatest impact month by the participated clinicians in Bangladesh, Japan and global survey. The volume of patient’s percentage was responded clinicians’ impression on their institution. *P*-value was < 0.0001 and significant. Bangladesh survey conducted in 2022 where Japan and global survey conducted in 2021 and 2020, respectively

Challenges to resume services to pre-COVID-19 level such Patient anxiety and fear limited (Bangladesh Survey: 80.7% vs Japan Survey: 67.4% vs Global Survey: 37.9%, *p* < 0.0001), loss of space due to COVID-19 (Bangladesh Survey: 63.9% vs Japan Survey: 34.7% vs Global Survey: 19.4%, *p* < 0.0001) were significantly high from the response of clinician of Bangladesh than Japan and global survey (Table 3). Comparing the usage of telemedicine, clinicians in Bangladesh used it significantly higher than those in Japan (Bangladesh Survey: 83.1% vs Japan Survey: 67.3% vs Global Survey: 78.6%, *p* < 0.0001) (Fig. 6). Providing care related to COVID-19, except for COVID-19 vaccination (Bangladesh Survey: 21.7% vs Japan Survey: 61.2%, *p* < 0.0001) and Sars-CoV-2 (Bangladesh Survey: 18.1% vs Japan Survey: 51.5%, *p* < 0.0001) was significantly higher in Bangladesh than Japan survey (Table 3). Except improved referral networks for complex patients and raising awareness on infectious disease control, other potential benefits of COVID-19 on hepatitis related care were significantly higher based on Bangladesh than Japan and global survey.

## Discussion

This is the first study to assess the impact of COVID-19 on hepatitis-related services in Bangladesh and compare the effect of COVID-19 in the developing country Bangladesh with the developed country Japan and the global situation.

Among all the services related to HBV and HCV diagnosis and treatment, all the clinicians reported some level of decline in Bangladesh survey. COVID-19 preventive measures such as strict lockdown initiated from March 2020 and extended till May 2020 and lifted with several conditions [28–31]. Again strict countermeasure was taken in June 2021 to August 2021 which termed as shutdown [28, 29, 31]. Those strict countermeasures hinder living and healthcare system of general population [22, 32, 33]. Even supply chain of hepatitis related services were heavily disrupted during greatest impact month of COVID-19 in Bangladesh. Comparing with the impression of clinicians in Japan survey and global survey, all the services severely declined in Bangladesh during the greatest impact month of COVID-19. Beside the Japan survey, comparing with other studies in developed countries and the answers of Bangladeshi clinicians, the level of decline reported was higher in Bangladesh than observed declines in other countries such as the Netherlands, Canada (British Columbia, Ontario), the USA, and China [34–36]. Furthermore, the decline reported by the clinicians in Bangladesh survey was greater than the decline reported in the European Association for the Study of the Liver (EASL) survey among the members [37]. The decline in all services related to HBV and HCV might hamper efforts to meet the set elimination goal and increase the disease burden in Bangladesh [2, 38, 39]. The availability of HBV-related facilities was wider compared to HCV in Bangladesh [12, 18, 40]. But interestingly, a significant difference in the disruption of HBV was higher compared to HCV treatment initiation. Further investigations required to identify the causal factors on more decline of HBV treatment initiation than that of HCV during COVID-19 pandemic.

Among all challenges to resuming services at the pre-COVID-19 levels, patient anxiety and fear of COVID-19 was reported highest in Japan [26]. However, clinicians in Bangladesh reported a significantly higher percentage of patients reporting anxiety or fear of COVID-19 than in Japan and global survey [25, 26]. Changes in infection control, such as increased use of a face mask or shield, gloves, and masks during patient encounters, were significantly higher in Bangladesh than in Japan among clinicians. Another interesting finding was 95.3% of clinicians involved in providing services related to COVID-19 in Bangladesh. It indicates that most of the clinicians in Bangladesh are flexible to provide care in any emergency or crisis.

Bangladeshi clinicians used telemedicine significantly more than Japanese clinicians and clinicians participated in global survey which was a positive impact. Additionally, beside telemedicine, a higher percentage of other strategies, such as the extension of the prescription period and advice to communicate with local doctors, could be utilized in other crisis situations in the future. Increased laboratory platforms for HBV and HCV testing, improved training in infectious diseases, and strengthening infectious disease control will be additional values of COVID-19 which must be capitalized on in the long run.

HBV and HCV are substantial public health problems with high mortality and morbidity rates that will require continuous and relentless dedication to reach goals for elimination [2, 3, 16]. COVID-19 has impacted funding and health care systems across the world, which is also clearly observed from this survey reported by clinicians. This study points to new gaps and challenges for such HBV-related services in the long run, which were more affected than HCV. So, this result suggests catching up on the missing gaps of HBV screening, treatment initiation, and patient monitoring. This result indicates that unscreened asymptomatic carriers would remain undiagnosed and untreated. This state could progress to liver cirrhosis, HCC, or even death. In addition, the decline and adversity of COVID-19 in developing countries were far greater than developed countries. That indicates the hepatitis care system in Bangladesh was constrained and it would be a bigger challenge after the post-pandemic period to overcome, including additional disease burden, to achieve the hepatitis elimination goal.

This study’s interpretation of the findings was subject to several limitations. First, there were a limited number of participants from each of the eight divisions. Second, clinicians may have based their perception of the experience at their institutions on several factors, including postponed clinic visits and patient volume reductions, which may not have been validated. Thirdly, comparisons may have been biased because the pandemic stage and survey period for the surveys in Bangladesh, Japan and global were not the same. Additionally, several aspects and questions were not possible to compare with global survey and Japan survey. However, we have asked the clinicians about the greatest impact month of COVID-19 based on their impressions and institutions.

## Conclusion

According to the response of clinicians, all the services related to HBV and HCV in Bangladesh were severely affected and higher than the findings in Japan and global survey. The constrained healthcare facilities and repeated preventive countermeasures of COVID-19 such as lockdown, shutdown and challenges such as anxiety and fear among patients of COVID-19 infection, loss of spaces were key reasons. However, usage of telemedicine and possible benefits of the COVID-19 response, including laboratory testing platform and improved training of clinicians in infectious disease testing and management should be utilized in the future viral hepatitis elimination program in Bangladesh.

### Supplementary Information

**Additional file 1:** **Supplementary Table 1.** Questionnaire for the survey participants.

**Additional file 2:** **Supplementary Figure 1.** The decline level of HCC related services in Bangladesh during highest impact month of COVID-19.

## Data Availability

All the data used and analyzed in the survey of this study is available from the corresponding author on reasonable request.

## Abbreviations

WHO: World Health Organization
HBV: Hepatitis B Virus
HCV: Hepatitis C Virus
COVID-19: Coronavirus disease 2019
SARS-CoV-2: Severe acute respiratory syndrome coronavirus 2
NLFB: National Liver Foundation of Bangladesh
JSH: Japan Society of Hepatology
PPE: Personal Protective Equipment
HCC: Hepatocellular carcinoma
SVR: Sustained Virological Response
