# Supplementary material for: The impact of COVID-19 on hepatitis B and C virus prevention, diagnosis, and treatment in Bangladesh compared with Japan and the global perspective
Source: BMC Health Serv Res. 2023 Oct 23;23:1137. doi: 10.1186/s12913-023-10138-x (PMC10594740; doi:10.1186/s12913-023-10138-x)
Supplement: Supplementary file 1 — Additional file 1: Supplementary Table 1. Questionnaire for the survey participants. [file 12913_2023_10138_MOESM1_ESM.docx]

**Supplementary Table 1**

**Questionnaire for the survey participants**

**Consent**

**I, thereby declared to understand the survey purpose and participant on the survey voluntarily with own willingness.**

No

Yes

| **No.** | **Question** | **Answer(s)/ Options/ Select/ comment** |
| --- | --- | --- |
| 1* | Occupation | Medical doctor / Others |
| 2* | Are you directly involved in testing, treatment, interventions care & prevention related to Hepatitis B virus/ Hepatitis C virus in Bangladesh |  |
| 3* | Your designation |  |
| 4* | Your specialty | Hepatologist/ Gastroenterologists/ Hepatobiliary & liver transplant surgeon/ others |
| 5* | District of Employment |  |
| 6* | What type of institution/facility are you affiliated with? | University Hospital/ Public Hospital/ medical collage/ Private Hospital/ Nonprofit liver organization/clinic/Health checkups center/ Government agency/ Company/ /Others |
| 7* | Are you head/ Professor/ Associate professor, Assistant professor, director of your department/ clinic/ hospital/ institution etc. regarding liver disease or hepatitis control at your facility? | Yes/ No |

**Section 02 (HBV and HCV related services)**

| **No.** | **Question** | **Answer(s)/ Options/ Select/ comment** |
| --- | --- | --- |
| 8 | (Impact of COVID-19 on hepatitis service delivery) Please indicate which product supply chains have been disrupted during the COVID-19 response? (Select all that apply) | HBV Vaccine/ HBV diagnosis/ HCV diagnosis/ HBV Treatment/ HCV Treatment/syringe-Injection equipment/ Awareness campaigns/Advocacy, Medication/ Hospital visits, Emergency care, Hepatic interventions, Follow up disruption, Medicine availability & supply. Decreased purchasing capacity of medicines, Others / Unknown/ None |
| 9 | Are disruptions in the supply chain on going? | Yes/ No/ Unknown/ NA |
| 10 | (Impact of COVID-19 on hepatitis service delivery) Have hepatitis-related program management meetings been deferred or cancelled? | Yes/ No/ NA |
| 11 | (Changes in patient volumes comparing a typical month pre-COVID to the month with the most substantive change during the epidemic, in your care setting what was the approximate decline) In number of persons screened for HBV? | None/ 1-25%/ 26-50%/ 51-75%/ 76-99%/ 100%/ Unknown/ NA |
| 12 | (Changes in patient volumes comparing a typical month pre-COVID to the month with the most substantive change during the epidemic, in your care setting what was the approximate decline) In number of persons receiving HBV confirmatory testing? | None/ 1-25%/ 26-50%/ 51-75%/ 76-99%/ 100%/ Unknown/ NA |
| 13 | (Changes in patient volumes comparing a typical month pre-COVID to the month with the most substantive change during the epidemic, in your care setting what was the approximate decline) In number of persons screened for HCV? | None/ 1-25%/ 26-50%/ 51-75%/ 76-99%/ 100%/ Unknown/ NA |
| 14 | (Changes in patient volumes comparing a typical month pre-COVID to the month with the most substantive change during the epidemic, in your care setting what was the approximate decline) In number of patients receiving HCV confirmatory tests? | None/ 1-25%/ 26-50%/ 51-75%/ 76-99%/ 100%/ Unknown/ NA |
| 15 | (Changes in patient volumes comparing a typical month pre-COVID to the month with the most substantive change during the epidemic, in your care setting what was the approximate decline) In number of persons initiated on HBV treatment? | None/ 1-25%/ 26-50%/ 51-75%/ 76-99%/ 100%/ Unknown/ NA |
| 16 | (Changes in patient volumes comparing a typical month pre-COVID to the month with the most substantive change during the epidemic, in your care setting what was the approximate decline) In number of persons initiated on HCV treatment? | None/ 1-25%/ 26-50%/ 51-75%/ 76-99%/ 100%/ Unknown/ NA |
| 17* | Did you have to extend the interval between hospital visits? | Yes/ No/ NA |
| 18 | (For the month with most substantive change during the covid-19 epidemic and for the current month, what were the trends in clinic visit deferrals and in the use of telemedicine?) For the month with the greatest COVID-19 impact, what percent of clinic visits for routine care were deferred? | None/ 1-25%/ 26-50%/ 51-75%/ 76-99%/ 100%/ Unknown/ NA |
| 19 | (For the month with most substantive change during the covid-19 epidemic and for the current month, what were the trends in clinic visit deferrals and in the use of telemedicine?) For the past month, what percent of clinic visits for routine care were deferred? | None/ 1-25%/ 26-50%/ 51-75%/ 76-99%/ 100%/ Unknown/ NA |
| 20 | Did you have to extend the prescription interval? | Yes/ No/ NA |
| 21 | During the response, for some or all patients did you Defer imaging? | Yes/ No/ NA |
| 22 | During the response, for some or all patients did you Defer lab testing for patients? | Yes/ No/ NA |
| 23* | During the response, for some or all patients did you have to defer HCC screening for patients? | Yes/ No/ NA |
| 24* | Did nutritional guidance for patients decrease? | Yes/ No/ NA |
| 25* | Did gastrointestinal endoscopy & intervention decrease? | Yes/ No/ NA |
| 26* | Did the number of liver biopsy hospitalizations decrease? | Yes/ No/ NA |
| 27* | Did the number of referrals for viral hepatitis patients from other departments in the hospital decrease? | Yes/ No/ NA |
| 28* | Did the liver disease lectures for patients decrease? | Yes/ No/ NA |
| 29* | Did the enlightenment activities by public lectures (awareness) decrease? | Yes/ No/ NA |
| 30 | (Changes in patient volumes comparing a typical month pre-COVID to the month with the most substantive change during the epidemic, in your care setting what was the approximate decline) In number of persons monitored for HBV treatment? | None/ 1-25%/ 26-50%/ 51-75%/ 76-99%/ 100%/ Unknown/ NA |
| 31 | (Changes in patient volumes comparing a typical month pre-COVID to the month with the most substantive change during the epidemic, in your care setting what was the approximate decline) In number of persons monitored for HCV treatment? | None/ 1-25%/ 26-50%/ 51-75%/ 76-99%/ 100%/ Unknown/ NA |
| 32 | (Changes in patient volumes comparing a typical month pre-COVID to the month with the most substantive change during the epidemic, in your care setting what was the approximate decline) In number of patients who receive HCV treatment? | None/ 1-25%/ 26-50%/ 51-75%/ 76-99%/ 100%/ Unknown/ NA |
| 33* | (Changes in patient volumes comparing a typical month pre-COVID to the month with the most substantive change during the epidemic, in your care setting what was the approximate decline) In number of patients who receive SVR12 for HCV treatment? | None/ 1-25%/ 26-50%/ 51-75%/ 76-99%/ 100%/ Unknown/ NA |
| 34* | (Changes in patient volumes comparing a typical month pre-COVID to the month with the most substantive change during the epidemic, in your care setting what was the approximate decline) In number of persons treated for drug addiction? | None/ 1-25%/ 26-50%/ 51-75%/ 76-99%/ 100%/ Unknown/ NA |
| 35* | (Changes in patient volumes comparing a typical month pre-COVID to the month with the most substantive change during the epidemic, in your care setting what was the approximate decline)　In number of patients who receive treatment for HCC? | None/ 1-25%/ 26-50%/ 51-75%/ 76-99%/ 100%/ Unknown/ NA |
| 36* | (Changes in patient volumes comparing a typical month pre-COVID to the month with the most substantive change during the epidemic, in your care setting what was the approximate decline)　In number of patients monitored for HCC treatment? | None/ 1-25%/ 26-50%/ 51-75%/ 76-99%/ 100%/ Unknown/ NA |

**Section 03 (Challenges and Mitigation Strategies)**

| **No.** | **Question** | **Answer(s)/ Options/ Select/ comment** |
| --- | --- | --- |
| 37 | What are the top challenges for resuming services to pre-COVID levels? | Patient anxiety or fear/ Limited staff available/ Inadequate PPE/ Loss of clinic space to COVID-19 response/ Loss of staff to COVID-19 response/ Supply shortage/ Loss of funding, with direction to COVID-19/ Others/ The level of medical services has not changed since pre-COVID/ I don't know |
| 38 | What type of telemedicine has been used? | Audio only/ Video via Phone/ Video via Computer/ Others /No |
| 39 | For the month with the greatest COVID-19 impact, what percent of clinic visits were conducted by telemedicine? | None/ 1-25%/ 26-50%/ 51-75%/ 76-99%/ 100%/ Unknown/ NA |
| 40 | For the past month, what percent of clinic visits were conducted by telemedicine? | None/ 1-25%/ 26-50%/ 51-75%/ 76-99%/ 100%/ Unknown/ NA |
| 41* | Besides telehealth, what other strategies have you or your care setting used to maintain or return HBV and HCV testing and treatment to typical volumes? | Extension of prescription period (long-term administration) / Referral to family doctor (reduction of burden on hospital visit) / providing medicines through courier services, Cooperation with industrial physician / Others /Nothing particular/ Don't know |

**Section 04 (Doctor’s Response to COVID-19)**

| **No.** | **Question** | **Answer(s)/ Options/ Select/ comment** |
| --- | --- | --- |
| 42 | How did infection control change among staff caring for persons tested or treated for hepatitis? (Select or enter value) | face mask, face shield routinely used during patients encounter/ Gloves, mask routinely used by staff during patients encounter/ Patient routinely check for COVID symptoms/Rigorous cleaning of surfaces/ face mask required of patients/ spacing of patients visit/ patient routinely assessed about fever via phone call or on arrival/ Others |
| 43* | Have your work activities changed in response to the emergence of COVID-19? | Yes/ No |
| 44* | Please indicate your role in testing, and providing clinical care for persons with possible COVID-19 (Select all that apply) | Triage outpatient /COVID-19 Vaccination / Sars-CoV-2 Testing/ Evaluate COVID-19 symptoms or sign and refer patients with severe disease/ Manage all aspects of care for patients with COVID-19/ Consultation on liver or Infectious disease management/ Others |
| 45* | For the month with the greatest COVID-19 impact, what percent of your time is spent on testing and providing clinical care for persons with possible COVID-19? | None/ 1-25%/ 26-50%/ 51-75%/ 76-99%/ 100% |
| 46* | For the past month, what percent of your time is spent on testing and providing clinical care for persons with possible COVID-19? | None/ 1-25%/ 26-50%/ 51-75%/ 76-99%/ 100% |

**Section 04 (Potential benefits on hepatitis)**

| **No.** | **Question** | **Answer(s)/ Options/ Select/ comment** |
| --- | --- | --- |
| 47 | As a result of the COVID-19 response, do you see any of the following as immediate or possibly future benefits to hepatitis testing and treatment? | Increased laboratory platforms for HBV and HCV testing/ Improved training of primary care in infectious disease testing and management/ Improved reporting of laboratory result/ Improved referral networks for complex patients/ Improved disease surveillance/ Improved contact tracing that could be used for hepatitis prevention/ Strengthening/ Raising awareness against infectious disease/ Others / Nothing particular/ Don't know |
| 48 | Please provide other strategies and comment |  |
| 49 | E-mail Address |  |

***Highlighted numbers were additional questions in Bangladesh perspective from global survey questionnaire**
