# Supplementary material for: The impact of COVID-19 on hepatitis B and C virus prevention, diagnosis, and treatment in Bangladesh compared with Japan and the global perspective
Source: BMC Health Serv Res. 2023 Oct 23;23:1137. doi: 10.1186/s12913-023-10138-x (PMC10594740; doi:10.1186/s12913-023-10138-x)
Supplement: Supplementary file 2 — Additional file 2: Supplementary Figure 1. The decline level of HCC related services in Bangladesh during highest impact month of COVID-19. [file 12913_2023_10138_MOESM2_ESM.pdf]

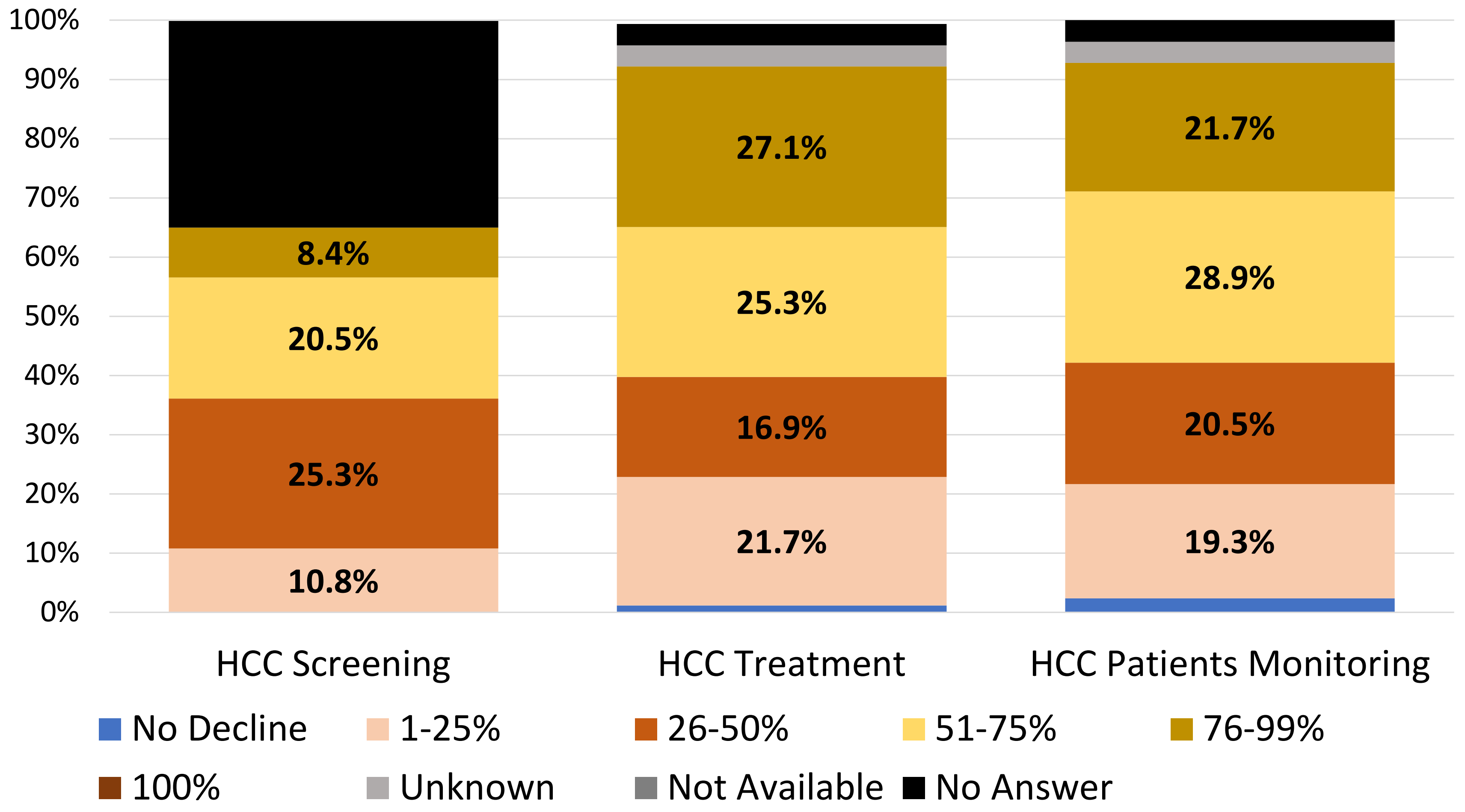

Supplementary Figure 1. The decline level of HCC related services in Bangladesh during highest impact month of COVID-19
